# Risk factors for first hospitalization due to meniscal lesions - a population-based cohort study with 30 years of follow-up

**DOI:** 10.1186/s12891-017-1886-5

**Published:** 2017-12-13

**Authors:** Tea Kontio, Markku Heliövaara, Harri Rissanen, Paul Knekt, Arpo Aromaa, Svetlana Solovieva

**Affiliations:** 1 University of Helsinki, Helsinki, Finland; 2 National Institute for Health and Welfare, Helsinki and Turku, Finland; 3 Finnish Institute of Occupational Health, 40, 00251 Helsinki, PB Finland

**Keywords:** Epidemiology, Knee, Meniscal lesion, Meniscal tear, Obesity, Risk factors

## Abstract

**Background:**

Meniscal lesions are among the most common injuries of the knee, yet limited epidemiologic data is available on their risk factors. We investigated the association of lifestyle factors and physical strenuousness of work on knee injuries with a focus on meniscal lesions.

**Methods:**

We examined a nationally representative sample of persons aged 30 to 59 years, who participated in a comprehensive health examination (the Mini-Finland Health Survey). Subjects without any injury or osteoarthritis in the knee joint at baseline (*n* = 4713) were subsequently followed via the National Hospital Discharge Register up to 30 years.

**Results:**

During the follow-up, 338 knee injuries were identified of which 224 were meniscal lesions. Obesity and regular leisure time physical exercise were associated with an increased risk of first hospitalization due to meniscal lesions (hazard ratio (HR) 1.62 and 95% confidence interval (CI) 1.06–2.48 and 1.53, 95% CI 1.05–2.23, respectively). The types of sports predicting the highest risk of meniscal lesions were ballgames, gymnastics and jogging. Physical strenuousness of work did not predict meniscal lesion. The hazard of other knee injury was increased among those reporting irregular or regular physical exercise at baseline (HR 1.64, 95% CI 1.03–2.64 and 1.88 CI 1.05–2.36, respectively). Smoking or alcohol intake were not associated with knee injuries.

**Conclusions:**

Better safety measures in high-risk sports and weight control would likely improve the prevention of meniscal lesions in populations.

## Background

Very little is known about the epidemiology of meniscal lesions, yet they are common [1, 2]. Meniscal lesions are often found together with osteoarthritis (OA) of the knee, and may therefore partly contribute to notable health care costs, disability and time lost from work associated with OA [2–6]. The rate of arthroscopic partial meniscectomies has steadily increased and has now become the most frequent orthopedic procedure [3, 7–9]. The association between meniscal lesions, their surgical treatment and elevated risk of osteoarthritis (OA) of the knee has been well documented [10–13]. Several meta-analyses and reviews have found no benefit in recovering from a degenerative meniscal tear for arthroscopic knee surgery compared with exercise in older patients [14, 15]. Recently, a new study was published reporting no clinically meaningful improvement in patient reported outcomes between patients with traumatic tears or degenerative tears after arthroscopic meniscectomy [16].

The incidence of a torn meniscus in the population is challenging to assess as these injuries may not be reported and can also be under-diagnosed [17]. In their study in Finnish male conscripts Kuikka et al. [18] suggested that the incidence of a meniscal tear would be 2.2 per 1000 person years. In an active North-American military population [19] the incidence was found to be as high as 8.27 per 1000 person years. Three other studies have estimated the incidence of a meniscal tear requiring surgical treatment to be 0.6–0.7 per 1000 persons annually [1, 5, 20].

Previous research concerning the risk factors of meniscal lesions is scarce [6]; most studies have been cross-sectional [21–23] and in longitudinal studies the follow-up periods have generally been rather short [5, 18, 19, 24]. Five prospective cohort studies have been published [3, 18, 25–27] of which only one was population-based [25]. The other studies have been mainly carried out in young athletes or members of the armed forces [19, 24, 28]. In these groups, the meniscal tears are often associated with a sports related event and would therefore be considered as traumatic [29]. In older patient groups, however, the tears are typically found together with osteoarthritis of the knee and degenerated meniscus [3, 4, 7, 25]. Previously identified risk factors of meniscal lesions comprise older age [3], obesity [6, 21] and work-related factors (kneeling, squatting) [30], knee malalignment and generalized OA [25] . Finally, most studies on meniscal tears have not included ambulatory visits nor knee injuries treated conservatively [5, 20, 23, 24]. The aim of our study was to assess lifestyle factors, such as obesity, leisure time physical exercise, smoking and alcohol intake as well as physical strenuousness of work for their prediction of knee injuries with an emphasis on those affecting the menisci.

## Methods

### Study population

A nationwide comprehensive health examination survey, the Mini-Finland Health Survey, was carried out by the Mobile Clinic of the Social Insurance Institution in 1978–1980. In brief, a population sample of 8000 subjects (3637 men and 4363 women) was drawn from the population register to represent Finnish adults aged 30 years or over. For practical reasons, a two-stage sampling design was used. At the first stage, 40 representative geographical areas were selected. At the second stage, a systematic sample of inhabitants was drawn from each area. The statistical efficiency of the sampling design proved sufficient. The implementation of the Mini-Finland Health Survey has been described in detail elsewhere [31] (https://www.thl.fi/en/web/thlfi-en/research-and-expertwork/population-studies/finnish-mobile-clinic/mini-finland-health-survey). Of the selected random sample, 5461 subjects were 30–59 years old.

**Fig. 1:**
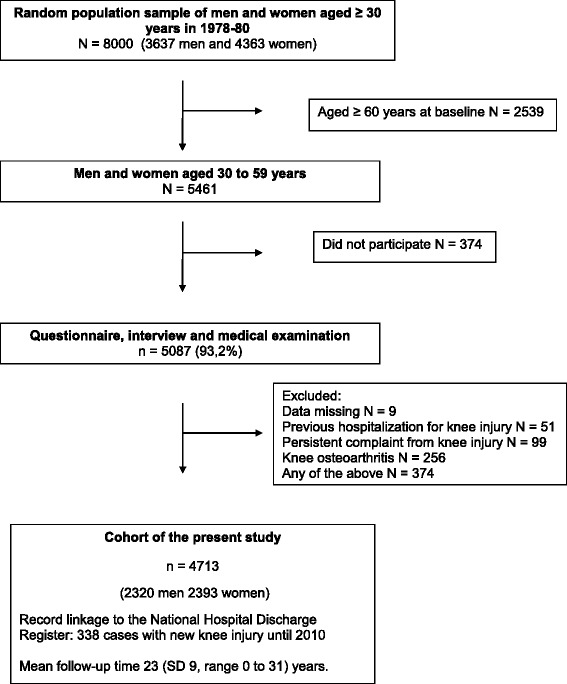
Flow chart of study sample formation

Altogether 93% of the sample (5087 subjects) participated in the initial screening phase, which comprised questionnaires, interviews, as well as laboratory and functional tests. This was followed by a medical examination. A physician classified all traumatic injuries based on the International Classification of Diseases, 8th edition, according to the information obtained from the medical history, symptom history and the physical examination. The subjects with a persistent injury or osteoarthritis of the knee or previous hospitalization due to knee injury (information drawn from the National Hospital Discharge Register) were excluded from the cohort of this study (Fig. 1). After these exclusions, there were 2320 men and 2393 women who comprised the present study population.

### Hospitalizations due to knee injuries

Information for the follow-up of knee injuries was drawn from the National Hospital Discharge Register using the subjects’ personal identification codes. The main outcome variable (hospitalization due to knee injuries) comprised all incident hospitalizations due to any traumatic knee injuries at any time between 1 January 1978 and 31 December 2010. The follow-up time in this study refers to the time from the baseline examination until the first admission to hospital for any knee injury, death, or the end of observation period, whichever came first. The follow-up time was counted in days.

In Finland, the 8th edition of the International Classification of Diseases (ICD) has been used before the year 1986, the 9th edition in 1986–1995, and the 10th edition from 1996 on. The following ICD codes were used to identify and classify the first knee injury: meniscal lesion 724,10; 7170A – 7179X; 8360A; 8361A; 8362A; M23.0 – M23.9; S83.2, fracture of distal femur, patella or proximal tibia 821,21; 821,31; 821,91; 822,00–822,90; 823,00; 823,10; 823,90; 8212A – 8213X; 8220A – 8221A; 8230A; 8231A; S72.4; S82.0; S82.1, luxation 836,00–836,91; 8363A – 8366B; S83.0; S83.1, distension, distortion or ligament rupture 844,00; 8440A – 8449X; S83.3 – S83.7.

### Potential risk factors

In the basic questionnaire filled in at the screening phase, leisure time physical exercise was inquired with the question: “How much do you move about and how hard do you exert yourself physically in your spare time?”. The response options were 1) only a little physical exercise, 2) physical exercise as part of other hobbies or irregularly, and 3) regular physical exercise. The participants who reported at least irregular exercise were asked to list the most common types of exercise they engaged in during summer and winter, at least four types for each season. We assumed that subjects with no or little physical exercise did not participate in the specific types of sports, therefore they were classified as “no exercise” in the further analyses. After the examination, the records were classified using a detailed list comprising codes for 90 types of exercise [32]. In this study, we chose to examine high impact sports (ballgames, gymnastics, jogging, alpine skiing) based on our hypothesis and previous literature, but also included some other common types of low impact sports in Finland at the time (skiing, bicycling, walking).

Both the baseline interview and the basic questionnaire elicited information on education, occupation and physical strenuousness of work. The level of education based on the home interviews was classified into three categories based on the years of education (<8 years (less than primary school), 8–12 years, (primary school plus lower or higher level secondary education), and >12 years (higher education, mostly studies or degrees at university level)). Physical strenuousness of work was classified into four categories, one of them including persons not at work at the time of interview. Other categories were light or mostly sedentary work, moderately heavy work including standing and some walking and heavy physical work. The basic questionnaire also inquired about average weekly consumption of beer, wine and strong beverages during the preceding month. The overall alcohol intake was calculated and expressed in grams of ethanol per week. Smoking history was obtained through standard questions at the interview. Standing height and weight were measured at the screening examination in light indoor clothing without shoes. Body mass index (BMI, weight/height^2^) was used as a measure of relative weight.

Clinical examinations were carried out by specially trained physicians following a standardized written protocol. Examination of the knee joint was carried out if any musculoskeletal disorder was suspected based on the screening (https://www.thl.fi/en/web/thlfi-en/research-and-expertwork/population-studies/finnish-mobile-clinic/mini-finland-health-survey). The standardized examination included estimations of limitations in the range of motion, tenderness, deformations, joint effusion and stability of the knee joint. The physicians made their final diagnoses based on clinical findings, knee symptoms, disease histories and related documentation applying uniform diagnostic criteria [33].

### Statistical analysis

Cox proportional hazards models were used to estimate the associations of possible risk factors and knee injury. Adjusted hazard ratios (HRs) with 95% confidence intervals (CI’s) were estimated. First, the effect of age and gender on outcome was explored. Second, the potential risk factors were included into the model in the following order: 1) BMI, 2) physical heaviness of work, 3) level of education and leisure-time physical activity and 4) smoking history and alcohol consumption. Only the final, mutually adjusted model (each variable adjusted for other variables in the model) is presented. Since the statistical efficiency of the study population proved sufficient in comparisons between design-based and model-based variance estimators (https://www.thl.fi/en/web/thlfi-en/research-and-expertwork/population-studies/finnish-mobile-clinic/mini-finland-health-survey), the sampling design was not considered in the statistical analysis. All the analyses were performed using the SPSS V23.0.

## Results

During the 120,868 person-years of follow-up, 338 subjects (147 men and 191 women) were diagnosed with a knee injury. A total of 224 persons (4.8%) had a meniscal lesion (97 men and 127 women) and the remainder (*N* = 114, 2.4%) had another type of knee injury.

The cumulative incidence of hospitalization due to meniscal lesions was sharply decreasing with baseline age (Table 1). Mean age at the time of the event (meniscal lesion or other knee injury) is shown in Table 2. For both outcomes, men had their hospitalization earlier than women. In the mutually adjusted model, meniscal lesions were more frequent in the younger population and among those engaged in regular leisure-time physical activity than others (Table 3). Regular physical activity carried a significant risk for both meniscal lesions and other knee injuries (HR 1.53, 95% Cl 1.05–2.23 and 1.88, 95% Cl 1.05–3.36), respectively. A higher BMI was an independent risk factor for contracting a meniscal lesion. Those not at work at baseline had an increased risk for other type of knee injury (HR 2.80, 95% Cl 1.47–5.34). Level of education, physical strenuousness of work, smoking history and alcohol consumption were not associated with either of the outcomes.

**Table 1:** Cumulative incidence (%) of hospitalization due to knee injury by baseline characteristics

		No hospitalization due to knee injury	Hospitalization due to meniscal lesion		Hospitalization due to other knee injury	
		N	N	%	N	%
Age group						
	30–39 years	1638	131	7.2	46	2.5
	40–49 years	1436	65	4.2	40	2.6
	50–59 years	1301	28	2.1	28	2.1
Gender						
	Men	2173	97	4.2	50	2.2
	Women	2202	127	5.3	64	2.7
Education						
	<8 years	2645	118	4.2	68	2.4
	8–12 years	1097	68	5.7	29	2.4
	>12 years	633	38	5.5	17	2.5
Physical strenuousness of work						
	Light	1171	65	5.1	28	2.2
	Moderate	1800	115	5.9	39	2.0
	Heavy	979	35	3.4	29	2.8
	Not at work	425	9	2.0	18	4.0
BMI (kg/m^2^)						
	< 25	2215	116	4.8	63	2.6
	25.0–29.9	1648	80	4.5	39	2.2
	≥ 30	512	28	5.1	12	2.2
Leisure time physical exercise						
	Little	1362	58	4,0	25	
	Irregular	2243	105	4,4	64	
	Regular	770	61	7,1	25	
Smoking						
	Never- smoker	2244	124	5.1	66	2.7
	Ex-smoker	901	47	4.9	20	2.1
	Current smoker	1230	53	4.0	28	2.1
Alcohol intake (g/wk)						
	0	1550	63	3.8	41	2.5
	1–49	2171	130	5.5	55	2.3
	≥ 50	649	31	4.4	18	2.6

**Table 2:** Mean age at the time of event

		Age (years)	SD (years)
Meniscal lesion	Men	56.2	10.9
	Women	58.5	9.0
Other injury	Men	55.4	12.9
	Women	62.0	14.1

**Table 3:** Adjusted hazard ratios (HR*) with 95% confidence intervals (CI) for hospitalization due to meniscal lesions or other knee injuries

Risk factor		N all	Meniscal lesion			Other knee injury		
			N cases	HR	95% Cl	N cases	HR	95% Cl
Age at baseline		4713	224	0.96	0.94–0.97	114	0.99	0.97–1.02
Gender	Men	2320	97	1		50	1	
	Women	2393	127	1.15	0.84–1.57	64	1.29	0.82–2.01
Education	<8 years	2831	118	1		62	1	
	8–12 years	1194	68	1.01	0.74–1.39	29	0.98	0.61–1.55
	>12 years	688	38	0.83	0.56–1.23	17	0.96	0.53–1.73
Physical strenuousness of work	Light	1264	65	1		28	1	
	Moderate	1954	115	1.20	0.86–1.64	39	0.90	0.55–1.49
	Heavy	1043	35	0.77	0.48–1.20	29	1.53	0.86–2.71
	Not at work	452	9	0.81	0.39–1.65	18	2.80	1.47–5.34
BMI	< 25.0	2394	116	1		63	1	
	25.0–29.9	1767	80	1.21	0.90–1.63	39	0.89	0.59–1.35
	≥ 30.0	552	28	1.62	1.06–2.48	12	0.96	0.51–1.81
Leisure time physical exercise	Little	1445	58	1		25	1	
	Irregular	2412	105	0.97	0.70–1.34	64	1.64	1.03–2.64
	Regular	856	61	1.53	1.05–2.23	25	1.88	1.05–3.36
Smoking	Never- smoker	2434	124	1		66	1	
	Ex-smoker	968	47	0.99	0.70–1.41	20	0.79	0.46–1.35
	Current smoker	1311	53	0.88	0.62–1.25	28	0.86	0.52–1.41
Alcohol intake	0 g/wk	1654	63	1		41	1	
	1–49 g/wk	2356	130	1.23	0.94–1.79	55	1.06	0.68–1.63
	50–249 g/wk	698	31	1.26	0.77–2.06	18	1.47	0.52–1.41

*Adjusted for other variables in the model

Ballgames, gymnastics and jogging were associated with an increased risk for meniscal lesions (HR 1.78, 95% Cl 1.18–2.72, 1.78, 95% Cl 1.14–2.77 and 1.69 95% CI 1.24–2.30, respectively). For alpine skiing, the hazard ratio was high (HR 2.31, 95% Cl 0.73–7.33), however due to small sample size it did not reach statistical significance. Bicycling, skiing and walking were not statistically significantly associated with meniscal lesions (HR 0.86, 95% Cl 0.62–1.19, 1.1, 95% Cl 0.83–1.42, 0.91, 95% Cl 0.68–1.22).

## Discussion

In the present population-based study we found that both baseline obesity and leisure time physical exercise were associated with an increased risk of first hospitalization due to meniscal lesions. Leisure time physical exercise was also a risk factor for other types of knee injuries. The types of sports carrying the highest risk of meniscal lesions were ballgames, gymnastics and jogging. In contrast, physical heaviness of work did not predict meniscal lesion. The risk of hospitalization due to meniscal lesions tended to be higher in women than men, however men had their first hospitalization approximately two years earlier than women.

Our findings regarding a detrimental effect of high BMI and certain types of sports on the knee are in accordance with previous studies. Both case-control [21, 23] and cohort [3, 18, 19] studies have reported obesity as a risk factor for meniscal lesion of the knee. Another population-based study [25] using knee MRI for the assessment of the outcome found obesity as a risk factor for medial meniscal extrusion but not for meniscal lesions in men and women aged 50–79 years. Due to different outcomes and populations, our results are not directly comparable with this or some other previous studies [18, 19, 34] . Participation in sports has been identified in younger populations as a risk factor for knee injuries in general [35, 36] and for meniscal tears in particular [21, 22, 37]. We found higher risks of any knee injury and meniscal lesion to be associated with regular leisure time physical exercise, although physical activity in the general population may not be directly comparable to the sports activity in a younger population. As expected [6, 34, 38], persons playing ballgames were at a higher risk for meniscal lesion compared with non-players. This is not surprising, considering the strains involved in rapid changes of direction, jumping and pivoting of the knee joint especially in soccer and basketball. In agreement with previous literature, [39] our findings suggested an increased risk for meniscal lesion in alpine skiers, however the sample size was small. Finally, we found a trend of women showing a greater risk for meniscal lesions than men, whereas in the other previously mentioned studies risk profiles were the opposite.

The menisci are of particular importance in the maintenance of the congruity and stability of the knee joint, in the protection against capsular or synovial impingement during motion and in the distribution of loads over the articular surface. With excess body weight, the delicate biomechanical mechanism is exposed to increasing loads during rotation, resulting in major strain and torque, making thereby the joint more susceptible to injury. It is also possible that due to compression, supply of nutrients to the menisci is reduced in obese persons which could, in part, increase perception of pain [40]. The role of inflammation resulting from obesity remains unclear in the development of meniscal and knee injury [41], yet the association has been shown for osteoarthritis [42, 43] and therefore could be linked to degenerative meniscal lesions. Further, it has been thought that traumatic and degenerative meniscal tears can be distinguished based on the onset of symptoms [3, 9, 29], type or location of the tear [44], age [3, 45, 46], presence of other degenerative changes in the knee joint [47], or presence of other co-occurring ligament tears and their types (mainly anterior cruciate ligament) [29]. These classifications are not clear-cut, and in many cases, the lesion can contain both traumatic and degenerative features. In their cross-sectional study, Baker et al. [21] attempted to distinguish risk factors between traumatic and degenerative tears on the basis of arthroscopy. Obesity was associated with degenerative tears and participation in sports (soccer in particular) was associated with acute traumatic tears.

A major strength of our study is that it is based on a large, nationally representative health examination survey of persons aged 31 years or over, and the participation rate of 90% was exceptionally high. Moreover, the follow-up period in our study extended up to 30 years. The study population was large enough to allow for adjustments for several relevant covariates. Furthermore, this study includes both conservatively and surgically managed meniscal injuries. The Finnish National Hospital Discharge Register - operating since 1967 - covers information on hospital admissions and discharges from every hospital in the country. This information includes the primary and secondary diagnoses according to the International Classification of Diseases, and it has been reported to be a reliable and accurate source of information [48, 49], although the specific diagnoses used in this study have not been validated. Because our cases included only those who were hospitalized, the results cannot be generalized to all meniscal lesions in the population.

For the follow-up, the first admission to the hospital because of knee injury was defined irrespective of the type of the injury (whether meniscal or other). This choice was made to simplify the study design and data analysis, and to focus on the first event. Thus, patients with recurrent meniscal injuries have not been taken into account. However, it is unlikely that this would have affected the main results of our study. At least in sports, combinations of injuries to cruciate ligaments and menisci are rather common. It is a limitation of our study that we could not distinguish sole meniscal tears and a combination of meniscal and ligamentous injuries. In our cohort design a given risk factor may have predicted a knee injury per se, hospitalization once the injury occurred, or both.

BMI measured at baseline predicted the risk for meniscal tears over 30 years later. Previous research suggests that obese persons in young adulthood tend to be obese also later in life [50], indicating a continuous exposure. Furthermore, the results concerning the increased risk of meniscal injury in the obese persons remained robust even after excluding the first 10 years, or the first 20 years of follow-up (data not shown).

Frequent leisure time physical exercise was a risk factor for both meniscal and other types of knee injuries. As our follow-up time was long, it is possible that there have been changes in the frequency of leisure time activity. It has, however, been previously shown for the Mini-Finland study population that those exercising actively at baseline are also active several years later [51]. We could not study all types of exercise or sports of interest, since many of them were rare at the time when our follow-up began more than 30 years ago. Furthermore, a severe knee injury is likely to influence sports activities later in life. It is also probable that a considerable share of the high impact sports is less frequent in populations later in life. We found no hazard for walking, bicycling or skiing activities that are certainly done throughout life.

We did not find any association between alcohol intake and knee injuries. The association between smoking and meniscal tears is not well known. In our study, smoking did not predict hospitalization due to meniscal lesions or other knee injuries. Neither was there an association between physical work load and meniscal lesions. Interestingly, a high risk for other knee injuries was observed for persons not working at baseline. These persons were either retired, unemployed or worked as housewives, thus they were a very heterogeneous population. A wide range of factors can underlie this finding.

In this cohort, we decided to exclude subjects over 60 years of age at baseline. After 60 years of age, the trauma profile is different and most of the meniscal lesions diagnosed are likely to be degenerative [3]. In our study, the mean age of contracting a meniscal lesion was around 50–60 years. Meniscal tears in special populations, such as athletes, occur at a relatively young age. In the systematic review by Snoeker et al. [6], meniscal tears were found to be more frequent in subjects over 60 years, however the tears were defined as degenerative due to exposure of longer time period. In a recent study by Pihl et al. [52] the authors found signs of early knee OA in patients undergoing arthroscopic knee surgery, further suggesting a concurrence of both meniscal lesions and knee OA already at the age of 40–50 years. However, a distinction between the types of tears, traumatic or degenerative, is not feasible in our study.

## Conclusions

The current study confirms the roles of obesity and regular physical exercise as independent risk factors for meniscal lesions and generalizes previous findings from clinical studies to the general population. Understandably, regular sports involving repetitive or acute injurious strain on the knee joint such as ballgames and gymnastics, involve higher risks for meniscal lesion than other types of sports. To prevent meniscal lesions, weight control is needed and more resources could be directed towards prevention of injuries in high risk sports.

## Abbreviations

BMI: Body mass index
CI: Confidence interval
HR: Hazard ratio
ICD: International Classification of Diseases
MRI: Magnetic resonance imaging
OA: Osteoarthritis
SPSS: Statistical Package for the Social Sciences
